# An Account of Diseases in an Eastern District of London, from Dec. 20, 1810, to Jan. 20, 1811

**Published:** 1811-02

**Authors:** 


### *An Account of Diseases in an Eastern District of London, from Dec.* 20, 1810, *to Jan.* 20, 1811.

| ACUTE DISEASES. | |
|---|---|
| Pneumonia - | 7 |
| Pleuritis - | 2 |
| Hæmorrhagia Intestinalis | 1 |
| Dysenteria - | 2 |
| Rheumatismus Acutus - | 3 |
| Dysuria - - | 3 |
| Prolapsus Vaginæ | 2 |
| Amenorrhœa - | 5 |
| Scrophula - - | 3 |
| Hypochondriasis Herpes | 12 |
| Rheumatismus Chronicus | 17 |

| CHRONIC DISEASES. | |
|---|---|
| Tussis - - - | 20 |
| Dyspnœa - - | 12 |
| Tussis cum Dyspnœa - | 10 |
| Catarrhus - - | 3 |
| Pleurodyne | 4 |
| Phthisis Pulmonalis - | 2 |
| Hœmoptysis - | 4 |
| Cephalalgia - | 3 |
| Gastrodynia - | 7 |
| Diarrhæa - - - | 4 |

| PUERPERAL DISEASES. | |
|---|---|
| Ephemera - - | 5 |
| Menorrhagia Lochialis | 4 |
| Dolor post partum | 3 |
| Enterodynia - | 6 |

| INFANTILE DISEASES. | |
|---|---|
| Aphthæ - - | 2 |
| Ophthalmia - | 5 |
| Herpes - - | 7 |
| Convulsio - - - | 3 |

In addition to this account it is sufficient to observe, that the state of disease during the last few weeks is very similar to that which was given in the last report. The different diseases of the lungs, which form a large portion of the list during the winter season, had then appeared, but in a more mild form. The extremely severe cold which has since prevailed, and which seems to have exceeded almost every thing which can be recollected for a considerable number of years, has produced a great aggravation of symptoms, amongst those who were before labouring under the complaint, and has given occasion to it amongst those in whose constitution there was a peculiar predisposition to it.

